# An efficient and provably secure key agreement scheme for satellite communication systems

**DOI:** 10.1371/journal.pone.0250205

**Published:** 2021-04-26

**Authors:** Yuanyuan Zhang, Zhibo Zhai

**Affiliations:** School of Computer Science, Hubei University of Technology, Wuhan, China; Xidian University, CHINA

## Abstract

Satellite communication has played an important part in many different industries because of its advantages of wide coverage, strong disaster tolerance and high flexibility. The security of satellite communication systems has always been the concern of many scholars. Without authentication, user should not obtain his/her required services. Beyond that, the anonymity also needs to be protected during communications. In this study, we design an efficient and provably secure key agreement scheme for satellite communication systems. In each session, we replace user’s true identity by a temporary identity, which will be updated for each session, to guarantee the anonymity. Because the only use of lightweight algorithms, our proposed scheme has high performance. Furthermore, the security of the proposed scheme is proved in the real-or-random model and the performance analysis shows that the proposed scheme is more efficient than some other schemes for satellite communication systems.

## Introduction

**Fig 1:**
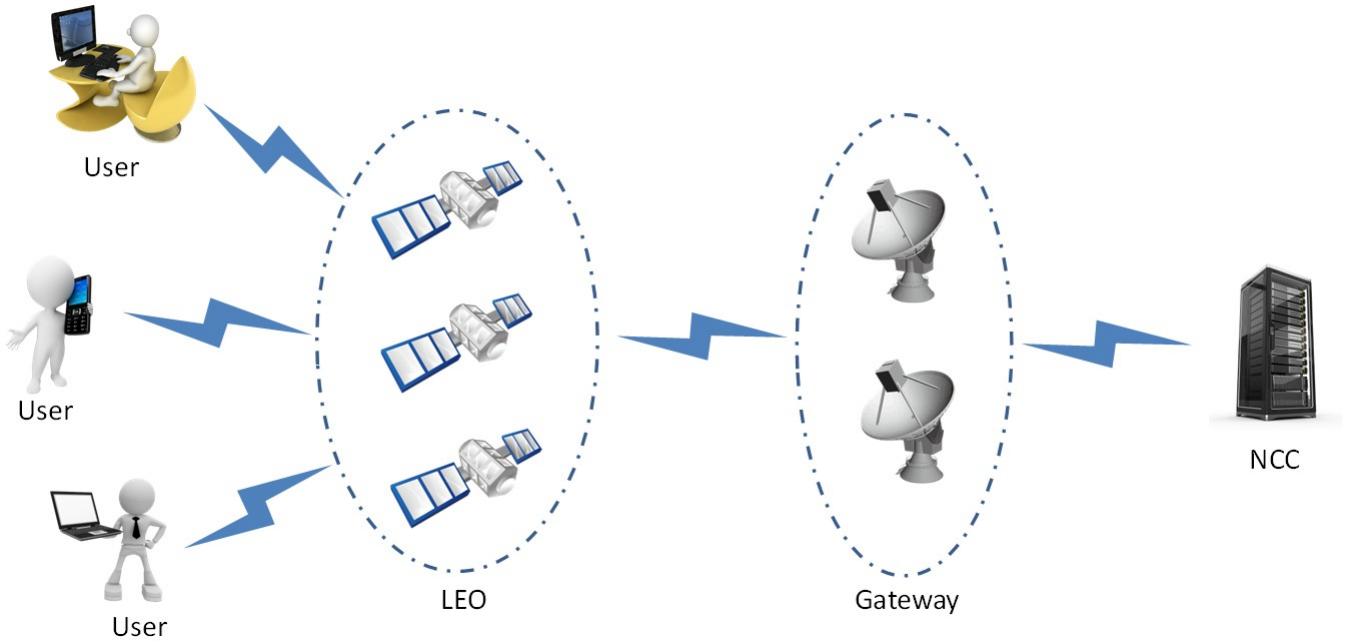
A *LEO* satellite communication system.

With the development of communication technology, satellite communication has played an important part in many industries, such as economy, politics, culture, military affairs, etc [1–3]. Compared with traditional satellite communication systems, the low-earth-orbit(*LEO*) satellite communication systems have the advantages of shorter transmission delay, higher efficiency, higher availability and higher cost performance. As shown in Fig 1, an *LEO* communication system is made up of mobile devices/users (*U*), gateways, *LEO* and the network control center (*NCC*). The most of entities in this *LEO* satellite communication system are connected wirelessly, the natural defects of the wireless communication bring great security risks, which will cause the system to be easily attacked by attackers.

In order to solve these problems, some low-Earth-orbit satellite communication protocols [4–7] have been proposed. In 1996, the first security satellite communication scheme was proposed by Cruickshank [8], but it cost too much computation. In 2003, Hwang *et al*. proposed an authentication scheme for mobile satellite communication system [9]. However, Chang *et al*. pointed out that their scheme couldn’t proved perfect forward secrecy [10]. In 2009, Chen *et al*. proposed a self-verification scheme and claimed there was no sensitive information stored in the verification [11]. But Lee *et al*. pointed out the user’s secret key is not secure in Chen *et al*.’s scheme when the hash value of user identity and another parameters are coprime numbers in 2011 [12]. In 2014, Zhang *et al*. proposed an improved authentication scheme [13]. But soon after, Qi *et al*. pointed out that Zhang *et al*.’s scheme was vulnerable to stolen-verifier attack and denial of service attack [14]. In 2017, Qi *et al*. proposed an enhanced authentication with key agreement scheme based on ECC(Elliptic Curves Cryptography), but the scheme has poor performance and some security defects. In this paper, we propose an enhanced scheme and prove that our scheme is secure under the real-or-random model.

In this context, an effective satellite communication scheme must possess the following characteristics to ensure the secrecy of normal operations in mobile satellite communication systems:

- Mutual authentication: *NCC* and user *U* can authenticate each other and generate a session key without being illegally obtained by the attacker.
- Perfect forward secrecy: if a session key or sensitive data is leaked to an attacker, he/she cannot obtain previous session keys from the preceding interception.
- Anonymity: the user’s identity is securely hidden and an attacker cannot derive user identity in any way.
- Resistance to stolen-verifier attacks: an attacker may break into servers and steal verification table from trusted servers. However, he or she cannot use the data in the verification table to launch any attack.
- Resistance to smart card loss attacks: if an attacker gets a legal user’s smart card in some way, he or she cannot derive sensitive data from it.
- Resistance to denial of service attacks: prevent attackers from occupying server resources illegally to ensure that the legal users can access the authentication server normally.
- Resistance to impersonation attacks: an attacker cannot attempt to communicate with a trusted server as a legitimate user and also cannot attempt to communicate with a legitimate user as a server.

The rest of this paper is organized as follows: We describe the detail of our proposed scheme in Section 2 and section 3 shows the security analysis of our scheme. Section 4 compares the security and the performance of our scheme with other related schemes. Finally, section 5 presents our conclusion.

## Our proposed scheme

In this section, we propose an efficient and provably secure key agreement scheme for satellite communication systems. Some notions in our scheme are shown in Table 1. In the proposed scheme, we abandon the traditional temporary identity verification table, and adopt a dynamic temporary identity table [15]. Usually, the traditional temporary identity table only stores temporary identity of this time. However, when an attacker intercepts messages returned from *NCC*, the data in *NCC*’s database has been updated and the data in the smart card has not been updated, so data inconsistency occurs between database and smart card. To solve this problem, we adopt a dynamic temporary identity table which consists of hash value of user’s identity, shared hash value, dynamic temporary identity of last time and dynamic temporary identity of this time(shown in Table 2).

**Table 1:** Notions in this paper.

*x*	Private key of *NCC*
*ID*_*u*_	Identity of user *U*
*T*_*id*_	Temporary identity
*PW*	Password of user *U*
*LEO*_*id*_	Identity of *LEO*
*h*(⋅)	A one-way hash function
⊕	XOR operation
||	Concatenation operation

**Table 2:** Dynamic verification table.

Hash value of user’s identity(*hID*)	Shared hash value (*hPW*)	Dynamic identity of last time(*T*_*ido*_)	Dynamic identity of this time(*T*_*id*_)
110101…101100	011011…101111	NULL	111001…000011
110101…101101	101110…010001	010110…110110	010101…110110
… …	… …	… …	… …
… …	… …	… …	… …
011011…010101	100010…110001	011101…000101	011101…110010

Our scheme contains the following phases: initialization phase, registration phase, login and authentication phase and password update phase. The details of our scheme are as follows.

### Initialization phase

*NCC* chooses a large prime *x* as long-term private key randomly and specifies a secure hash algorithm *h*(⋅). In the meantime, *NCC* creates a table in the databases. The table stores four data for each legitimate user. Two of these data are the hash values which used to authenticate the identity. The remaining two are used to store user’s dynamic *ID*. One *ID* is for last time, the other one is for this time. If a user fails to authenticate with the this time *ID*, he will try to re-authenticate with the last time *ID*.

### Registration phase

**Fig 2:**
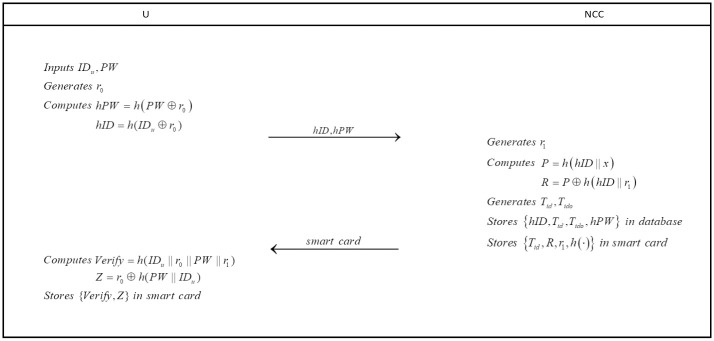
Registration phase.

To be a legal user, he/she must submit his/her registration request to *NCC* (see in Fig 2).

**step 1**: With the permission of *NCC*, user *U* inputs *ID*_*u*_ and *PW* chosen by himself/herself. After that, user *U* generates a random number *r*_0_ and computes

$$\begin{array}{c} hPW=h(PW⊕r_{0})\\ hID=h(ID_{u}⊕r_{0}). \end{array}$$

Next, user *U* sends {*hID*, *hPW*} as registration request to *NCC* in a secure channel.

**step 2**: After receiving registration message, *NCC* generates a random number *r*_1_ and computes

$$\begin{array}{c} P=h(hID||x)\\ R=P⊕h(hID||r_{1}) \end{array}$$

with *NCC*’s private key *x* and registration message. Next, *NCC* generates two temporary identity *T*_*id*_, *T*_*ido*_ and initializes *T*_*ido*_ to null. After that, *NCC* stores {*hID*, *T*_*id*_, *T*_*ido*_, *hPW*} in the database. Then, *NCC* writes {*T*_*id*_, *R*, *r*_1_, *h*(⋅)} into a smart card.

**step 3**: *NCC* delivers the smart card to user *U* in a secure channel.

**step 4**: User *U* computes

$$\begin{array}{cc} Verify & =h(ID_{u}||r_{0}||PW||r_{1})\\ Z & =r_{0}⊕h(PW||ID_{u}) \end{array}$$

with the data stored in smart card. Then user *U* writes {*Verify*, *Z*} into the smart card.

### Login and authentication phase

**Fig 3:**
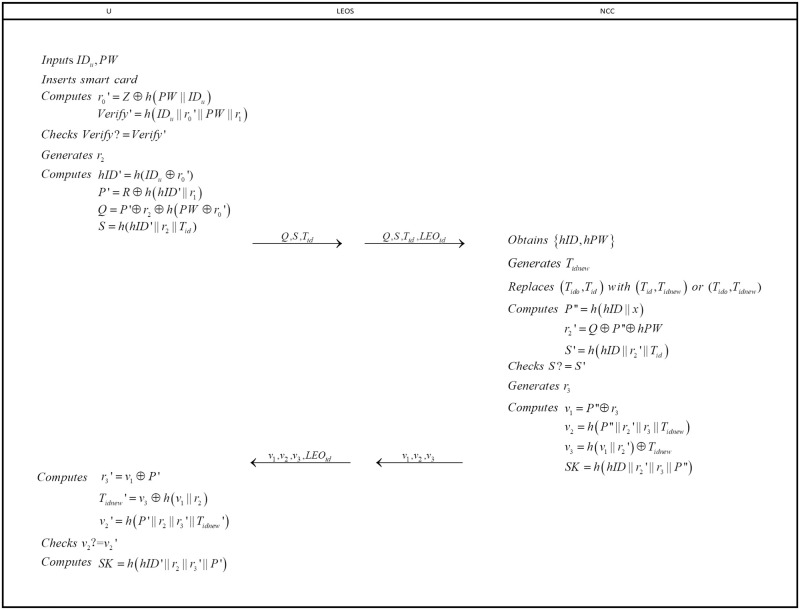
Login and authentication phase.

Login and authentication phase are indispensable steps for user to get services from server. The following operations are intended to achieve the goal (see in Fig 3).

**step 1**: User *U* inserts his/her smart card into card reader and inputs *ID*_*u*_ and *PW*. Then, *U* computes

$$\begin{array}{cc} {r_{0}}^{\prime } & =Z⊕h(PW||ID_{u})\\ Verify^{\prime } & =h(ID_{u}||{r_{0}}^{\prime }||PW||r_{1}) \end{array}$$

and verifies whether *Verify* = *Verify*′ is true. If not, the session will be broken off. Otherwise, user *U* generates a number *r*_2_ and computes

$$\begin{array}{cc} hID^{\prime } & =h(ID_{u}⊕{r_{0}}^{\prime })\\ P^{\prime } & =R⊕h(hID^{\prime }||r_{1})\\ Q & =P^{\prime }⊕r_{2}⊕h\left(PW⊕{r_{0}}^{\prime }\right)\\ S & =h(hID^{\prime }||r_{2}||T_{id}). \end{array}$$

Then, user *U* sends {*Q*, *S*, *T*_*id*_} to *LEO* in a public channel.

**step 2**: *LEO* receives the login request from *U* and forwards the login request {*Q*, *S*, *T*_*id*_, *LEO*_*id*_} to *NCC*.

**step 3**: After receiving the login request from *LEO*, *NCC* begins to match *T*_*id*_ in the dynamic verification table(Table 2). Firstly, *NCC* searches the column of *Dynamic identity of this time*(*T*_*id*_). If there is a value equals to *T*_*id*_, *NCC* extracts the corresponding *hID*, *hPW* and chooses a new temporary identity *T*_*idnew*_. Then *NCC* replaces (*T*_*ido*_, *T*_*id*_) with (*T*_*id*_, *T*_*idnew*_). Else, *NCC* keeps searching the column of *Dynamic identity of last time*(*T*_*ido*_) in the verification table to see if there is a value equals to *T*_*id*_. If so, *NCC* extracts the corresponding *hID*, *hPW*, chooses a new temporary identity *T*_*idnew*_ and replaces (*T*_*ido*_, *T*_*id*_) with (*T*_*ido*_, *T*_*idnew*_). If *NCC* cannot match *T*_*id*_ in the dynamic verification table, it will reject the login request.

**step 4**: After completion of matching, *NCC* computes

$$\begin{array}{cc} P^{\prime \prime } & =h(hID||x)\\ {r_{2}}^{\prime } & =Q⊕P^{\prime \prime }⊕hPW\\ S^{\prime } & =h(hID||{r_{2}}^{\prime }||T_{id}) \end{array}$$

and checks whether *S* and *S*′ are equal. If not, *NCC* breaks off the session. Else, *NCC* generates a random number *r*_3_ and computes

$$\begin{array}{cc} v_{1} & =P{}^{\prime \prime }⊕r_{3}\\ v_{2} & =h(P^{\prime \prime }||{r_{2}}^{\prime }||r_{3}||T_{idnew})\\ v_{3} & =h(v_{1}\left|\right|{r_{2}}^{\prime })⊕T_{idnew}\\ SK & =h(hID||{r_{2}}^{\prime }||r_{3}||P^{\prime \prime }). \end{array}$$

*SK* will be using as the session key between user *U* and *NCC*. After that, *NCC* delivers {*v*_1_, *v*_2_, *v*_3_, *LEO*_*id*_} to *LEO*.

**step 5**: *LEO* forwards the message {*v*_1_, *v*_2_, *v*_3_} from *NCC* to user *U*.

**step 6**: After receiving {*v*_1_, *v*_2_, *v*_3_} from *LEO*, user *U* computes

$$\begin{array}{cc} {r_{3}}^{\prime } & =v_{1}⊕P^{\prime }\\ {T_{idnew}}^{\prime } & =v_{3}⊕h(v_{1}\left|\right|r_{2})\\ {v_{2}}^{\prime } & =h(P^{\prime }||r_{2}||{r_{3}}^{\prime }||{T_{idnew}}^{\prime }) \end{array}$$

and compares whether *v*_2_ and *v*_2_′ are equal. If verification is failed, the session will be broken off. Else, user *U* computes the session key *SK* = *h*(*hID*′||*r*_2_||*r*_2_′||*P*′).

### Password update phase

**Fig 4:**
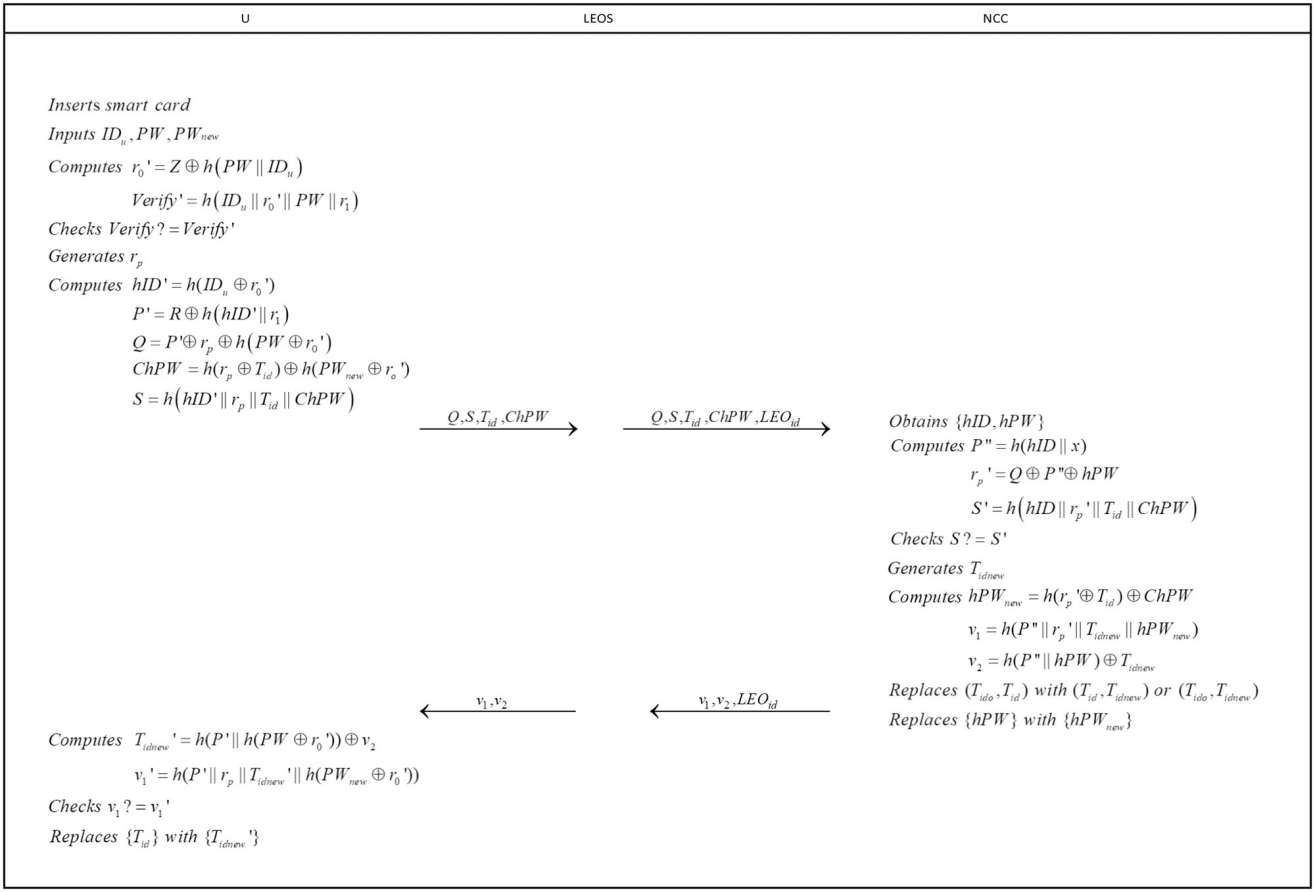
Password update phase.

To enhance security, users are advised to update their passwords at set intervals. To complete the process, user *U* will perform the following steps(see in Fig 4).

**step 1**: User *U* inserts the smart card and inputs *ID*_*u*_,*PW*,*PW*_*new*_ where *PW*_*new*_ is the new password chosen by himself/herself. Then user *U* computes

$$\begin{array}{cc} {r_{0}}^{\prime } & =Z⊕h(PW||ID_{u})\\ Verify^{\prime } & =h(ID_{u}||{r_{0}}^{\prime }||PW||r_{1}). \end{array}$$

After that, user *U* checks whether *Verify* equals to *Verify*′. If not, user *U* breaks off the session. Else, user *U* generates a random number *r*_*p*_ and computes

$$\begin{array}{cc} hID^{\prime } & =h(ID_{u}⊕{r_{0}}^{\prime })\\ P^{\prime } & =R⊕h(hID^{\prime }||r_{1})\\ Q & =P^{\prime }⊕r_{p}⊕h\left(PW⊕{r_{0}}^{\prime }\right)\\ ChPW & =h\left(r_{p}⊕T_{id}\right)⊕h\left(PW_{new}⊕{r_{0}}^{\prime }\right)\\ S & =h(hID^{\prime }||r_{p}||T_{id}||ChPW) \end{array}$$

After that, user *U* sends {*Q*, *S*, *T*_*id*_, *ChPW*} to *LEO*.

**step 2**: *LEO* forwards the message received form user with *LEO*_*id*_ to *NCC*.

**step 3**: Upon receiving the message, *NCC* executes the step 3 in Login and authentication phase to obtain *hID* and *hPW*. Then *NCC* computes

$$\begin{array}{cc} P^{\prime \prime } & =h(hID||x)\\ {r_{p}}^{\prime } & =Q⊕P^{\prime \prime }⊕hPW\\ S^{\prime } & =h(hID||{r_{p}}^{\prime }||T_{id}||ChPW) \end{array}$$

and checks whether *S* equals to *S*′. If not, *NCC* aborts the session. Else, *NCC* generates a new temporary identity *T*_*idnew*_ and computes

$$\begin{array}{cc} hPW_{new} & =h({r_{p}}^{\prime }⊕T_{id})⊕ChPW\\ v_{1} & =h(P^{\prime \prime }||{r_{p}}^{\prime }||T_{idnew}||hPW_{new})\\ v_{2} & =h(P^{\prime \prime }\left||hPW\right)⊕T_{idnew}. \end{array}$$

Next, *NCC* replaces (*T*_*ido*_, *T*_*id*_, *hPW*) with (*T*_*id*_, *T*_*idnew*_, *hPW*_*new*_) or (*T*_*ido*_, *T*_*idnew*_, *hPW*_*new*_). Then, *NCC* sends message {*v*_1_, *v*_2_, *LEO*_*id*_} to the *LEO*.

**step 4**: Upon receiving the message {*v*_1_, *v*_2_, *LEO*_*id*_}, *LEO* forwards {*v*_1_, *v*_2_} to *U*.

**step 5**: After receiving the message, user *U* computes

$$\begin{array}{cc} {T_{idnew}}^{\prime } & =h(P^{\prime }||h\left(PW⊕{r_{0}}^{\prime }\right))⊕v_{2}\\ {v_{1}}^{\prime } & =h(P^{\prime }||r_{p}||{T_{idnew}}^{\prime }||h\left(PW_{new}⊕{r_{0}}^{\prime }\right)) \end{array}$$

and checks whether *v*_1_ equals to *v*_1_′. If holds, replaces {*T*_*id*_} with {*T*_*idnew*_′} in the smart card.

## Security analysis

In this section, we will show that our scheme is provably secure in the real-or-random model.

### Security model

In 2005, Abdalla *et al*. introduced a new security model for two-party password-based authenticated key exchange scheme [16]. Based on their real-or-random model, we can prove the security of our scheme. In the model, there are two types of participants, user *U* and network control center *NCC*, respectively. *U*_*i*_ denotes the *i*_*th*_ instance of *U*. The adversary *A*, which is abstracted as a probabilistic polynomial time Turing Machine, interacts with other participants through a bounded number of queries which model the capabilities of the adversary in an actual attack. The ability of adversary *A* is defined by the following queries.

*Excute*(*U*_*i*_, *NCC*): Return the messages transmitted between *U*_*i*_ and *NCC* in their last key agreement conversation. This query models the eavesdropping attack.

*Send*(*U*_*i*_/*NCC*, *m*): After receiving message *m* sent by *A*, *U*_*i*_/*NCC* generates a corresponding message for *m* and outputs it as the result of this query. This query models the active attacks such as replay attack, impersonation attack and so on.

*CorruptSC*(*U*_*i*_): Return the current data stored in *U*_*i*_’s smart card. This query models the smart card lost attack.

*CorruptDB*(*NCC*): Return the current data stored in *NCC*’s database. This query models the insider attack and the stolen verifier attack.

*Test*(*U*_*i*_/*NCC*): The semantic security of the session key is simulated by flipping an unbiased coin. The query returns a random binary of the same size of session key if *b* = 0 or the session key between *U*_*i*_ and *NCC* if *b* = 1. The adversary can ask only one time of *Test* query.

*H*(*x*): This is a hash query. If a record (*x*, *h*) exists in the hash list, *h* is returned. Otherwise, return a uniformly random string *h* and store (*x*, *h*) in the table.

*Semantic security*: Providing the above-mentioned queries, the adversary *A* may interact with the participants to help him/her verify the value of *b*. If he/she can guess correctly, the scheme fails to provide semantic security. Let *Succ* denotes the event that *A* wins. *A* has an advantage

$$\begin{array}{c} Adv^{ake}\left(A\right)=\left|Pr\left[Succ\right]-\frac{1}{2}\right| \end{array}$$

in breaking the semantic security of the scheme. If *Adv*^*ake*^(*A*) is negligible, the scheme is secure under the real-or-random model.

### Security proof

Theorem 1: Let *S*_*ID*_ and *S*_*PW*_ be uniformly distributed dictionary of user identity and password, respectively. |*S*_*ID*_| and |*S*_*PW*_| denoted the size of *S*_*ID*_ and *S*_*PW*_. |*H*| denotes the range space of the hash function. Beyond that, we denote *q*_*h*_ and *q*_*s*_ to represent the number of *H*(*x*) oracle queries and the total number of queries executed by *A*. Then, we have

Advake(A)≤qh22|H|+max{qs|SID|⋅|SPW|,qs2k}

Proof: We define several attack games from game *Gm*_0_ to game *Gm*_3_. For each game *Gm*_*i*_, *Succ*_*i*_ denotes the event that *A* has successfully guessed the bit *b* in the test session. The games are listed as follows:

Game *Gm*_0_: This game models the real attack by the adversary. We have

$$\begin{array}{c} Adv^{ake}\left(A\right)=\left|Pr\left[Succ_{0}\right]-\frac{1}{2}\right| \end{array}$$ (1)

Game *Gm*_1_: To increase the advantage of success, *A* launches an eavesdropping attack by querying the *Excute*(*U*_*i*_, *NCC*) oracle. Since the session key *SK* is computed by *hID*, *r*_2_,*r*_3_ and *P*, *A* tries to obtain these values from the messages transmitted in the public channel. We know that *r*_2_ = *Q* ⊕ *P* ⊕ *hPW*, *r*_3_ = *v*_1_ ⊕ *P* and *P* = *h*(*hID*||*x*) = *R* ⊕ *h*(*hID*||*r*_1_). The *hID* is concealed in the hash function. Thus, *A* cannot get the values of *hID*, *r*_2_, *r*_3_ and *P*. In this game, the *Excute*(*U*_*i*_, *NCC*) query dose not provide any advantage compared to game *Gm*_0_ and we have

$$\begin{array}{c} Pr\left[Succ_{0}\right]=Pr\left[Succ_{1}\right] \end{array}$$ (2)

Game *Gm*_2_: In this game, we add the *send* query to simulate an active attack. In order to pass the authentication, *A* must use *H*(*x*) query to fabricate messages. No collisions will be found in the input while querying *H*(*x*) oracle, because every message contains some different random numbers. By the birthday paradox, we can get

Pr[Succ2]−Pr[Succ1]≤qh22|H| (3)

Game *Gm*_3_: We transfer game *Gm*_2_ to this game by adding the *CorruptSC*(*U*_*i*_) query or the *CorruptDB*(*U*_*i*_) query.

Case 1: The adversary asks the *CorruptSC*(*U*_*i*_) query. Then he/she can extract {*T*_*id*_, *R*, *r*_1_, *Verify*, *Z*} stored in the user *U*_*i*_’s smart card. For the adversary, *hID* = *h*(*ID*_*u*_ ⊕ *r*_0_) = *h*(*ID*_*u*_ ⊕ *Z* ⊕ *h*(*PW*||*ID*_*u*_)), *r*_2_ = *Q* ⊕ *P* ⊕ *hPW* = *Q* ⊕ *R* ⊕ *h*(*hID*||*r*_1_) ⊕ *h*(*PW* ⊕ *Z* ⊕ *h*(*PW*||*ID*_*u*_)), *r*_3_ = *v*_1_ ⊕ *P* = *v*_1_ ⊕ *R* ⊕ *h*(*hID*||*r*_1_) and *P* = *h*(*hID*||*x*) = *R* ⊕ *h*(*hID*||*r*_1_). So *A* tries a dictionary attack with the possible identity and password of the user in *S*_*ID*_ and *S*_*PW*_. Since the scale of the dictionary is |*S*_*ID*_| and |*S*_*PW*_|, the adversary need to guess the correct values of *U*’s identity and password simultaneously. In this case, the probability of a successful dictionary attack is negligible. So, we have

Pr[Succ3]−Pr[Succ2]≤qs|SID|⋅|SPW| (4)

Case 2: The adversary *A* asks the *CorruptDB*(*NCC*) query. Then he/she can extract {*hID*, *T*_*id*_, *T*_*ido*_, *hPW*} stored in *NCC*’s database. For the adversary, *r*_2_ = *Q* ⊕ *P* ⊕ *hPW* = *Q* ⊕ *h*(*hID*||*x*) ⊕ *hPW*, *r*_3_ = *v*_1_ ⊕ *P* = *v*_1_ ⊕ *h*(*hID*||*x*) and *P* = *h*(*hID*||*x*). So *A* tries a dictionary attack with the possible private key of *NCC*. So, we have

Pr[Succ3]−Pr[Succ2]≤qs2k (5)

where *k* is the security parameter.

The adversary *A* can choose case 1 or case 2 as the last game *Gm*_3_. From game *Gm*_0_ to game *Gm*_3_, all the oracles are simulated and *A* has no choice but querying the *Test* query and guessing the bit *b* in the last game. Therefore,

$$\begin{array}{c} Pr\left[Succ_{3}\right]=\frac{1}{2} \end{array}$$ (6)

Combining (1)-(6), we can derive

Advake(A)≤qh22|H|+max{qs|SID|⋅|SPW|,qs2k}

The advantage for an adversary to guess the correct session key is negligible since |*H*|,|*S*_*ID*_| ⋅ |*S*_*PW*_| and 2^k^ are beyond the polynomial time. So our scheme can provide semantic security in the real-or-random model.

### Other security features

In this section, we will analyze the security and practicability of our scheme [17–20].

*Provide credible mutual authentication*: In login and authentication phase, *NCC* can authenticate user *U* only when user *U* submits correct identity *ID*_*u*_, password *PW* which can satisfy the equation *S*′ = *h*(*hID*||*r*_2_′||*T*_id_) = *S*. An attacker cannot falsify valid *S* and *Q* to pass *NCC*’s authentication without the correct identity and password. User *U* can authenticate *NCC* only when the user receives the correct message {*v*_1_, *v*_2_, *v*_3_} which can satisfy the equation *v*_2_′ = *h*(*P*′||*r*_2_||*r*_3_′||*T*_*idnew*_′) = *v*_2_. But an attacker cannot counterfeit the correct message {*v*_1_, *v*_2_, *v*_3_} without *P*. Thus, our scheme provides credible mutual authentication.

*Provide perfect forward secrecy*: Forward secrecy means an attacker cannot obtain the past session keys even he/she has got *NCC*’s private key, users’ password and users’ identity. In our scheme, the session key *SK* = *h*(*hID*||*r*_2_||*r*_3_′||*P*′) is established with the user’s identity, the random number *r*_2_, *r*_3_ and the hash value *P* = *h*(*hID*||*x*). Even if an attacker obtains those sensitive data, he/she cannot obtain other session keys because he/she cannot derive *r*_2_ = *P* ⊕ *hPW* ⊕ *Q* without user *U*’s corresponding *hPW*. Therefore, our scheme can provide forward secrecy.

*Provide anonymity*: Some traditional schemes often transmitted users identity *ID*_*u*_ in the public channel, which may lead to leakage of identity information, obviously those schemes can not provide anonymity. There are also some schemes claim that they can provide anonymity. But their identities usually can be guessed by attackers. In our scheme, we transmit temporary identity *T*_*id*_ instead of user’s identity *ID*_*u*_, and the temporary identity will be updated after each session. And the attacker cannot guess *ID*_*u*_ from *R* = *P* ⊕ *h*(*hID*||*r*_1_), *Verify* = *h*(*ID*_*u*_||*r*_0_||*PW*||*r*_1_), *Z* = *r*_0_ ⊕ *h*(*PW*||*ID*_*u*_) or other equations. So our scheme can provide anonymity.

*Resist stolen-verifier attack*: Stolen-verifier attack means an insider attacker may steal the data in the database, and he can derive users’ password or impersonate legal users to send legitimate login requests. In our scheme, the database stores {*hID*, *T*_*ido*_, *T*_*id*_, *hPW*}, if the insider attacker steals these data, he/she cannot derive users’ password *PW* without the random number *r*_0_. In addition to this, the insider attacker cannot impersonate legal users to send legitimate login requests *Q* = *P*′ ⊕ *r*_2_ ⊕ *h*(*PW* ⊕ *r*_0_′) and *S* = *h*(*hID*||*r*_2_||*T*_*id*_) to *NCC* without the password *PW*, the secret data *P* and the random number *r*_0_. Therefore, our scheme can resist the stolen-verifier attack.

*Resist smart card loss attack*: Assume an attacker gets user *U*’s smart card and extracts the parameters {*T*_*id*_, *R*, *r*_1_, *h*(⋅), *Verify*, *Z*} from it and the attacker also intercepts the communication message between user *U* and *NCC*. But these parameters do not help him/her perform any attacks without the user’s password *PW* and identity *ID*_*u*_. Thus, our scheme can resist smart loss attack.

*Resist denial-of-service attack*: In login and authentication phase, after *NCC* authenticating user *U*, an attacker may intercept and modify the message {*v*_1_, *v*_2_, *v*_3_} which is forwarded from *NCC* to *U*. Obviously, the modified message cannot pass the authentication of user *U* and the updating of temporary identity will be inconsistent between *NCC* and user *U* which must lead to the denial-of-service attack. To avoid that, our scheme adopts dynamic temporary identity(see in Table 2), and *NCC* stores the temporary identity both of this time and last time. Even the update is inconsistent, the legal user also can login successfully next time with the old temporary identity. Thus, our scheme can resist denial-of-service attack.

*Resist impersonation attack*: Impersonation attack means that an attacker impersonates a legal user to login *NCC* or impersonates *NCC* to communicate with a legal user. In our scheme, if the attacker intends to impersonate a legal user *U*, he/she must computes *Q* = *P*′ ⊕ *r*_2_ ⊕ *h*(*PW* ⊕ *r*_0_′) and *S* = *h*(*hID*||*r*_2_||*T*_*id*_). However, *Q* and *S* are established with the secret data *P*, the temporary identity *T*_*id*_, the password *PW* and the identity *ID*_*u*_. All of those data are secret, so the attacker cannot impersonate a legal user. On the other hand, even if the attacker obtains the temporary identity of the user, he/she cannot know *P*′ = *h*(*hID*||*x*) without *NCC*’s private key *x*. So the attacker cannot masquerade as *NCC* without *P* to compute the correct message {*v*_1_, *v*_2_, *v*_3_}. Therefore, our scheme can resist impersonation attack.

## Security and performance comparison

In this section, we evaluate the performance of our proposed scheme with other three related schemes [14, 21, 22]. The computational cost in the login and authentication phase is compared in detail and the security features of these schemes are also analyzed.

In the proposed scheme, the traditional verification table is improved to form a dynamic one which can resist the denial-of-service attack. In addition, the value stored in the smart card is useless for the attacker to launch any attack. Even if the smart card is lost, user information will not be leaked out. As for the transmitting messages, we transmit user’s temporary identity *T*_*id*_ replace user’s true identity *ID*_*u*_. Thus, anonymity has been achieved.

As shown in Table 3, the related schemes [14, 21, 22] have some design flaws and cannot satisfy all the security features. Qi *et al*.′*s* scheme [14] suffers from smart card loss attack, denial-of-service attack, off-line guessing attack, replay attack. In addition to this, their scheme also cannot provide forward secrecy and anonymity. Lin *et al*.′*s* scheme [21] satisfies most of the security requirements, where as they ignore the smart card loss attack. In Mo *et al*.′*s* scheme [22], they cannot resist stolen-verifier attack, smart card loss attack, impersonation attack and cannot provide forward secrecy. Compared with the related works, our scheme can resist most of the known attacks and provide password update service for users to choose.

**Table 3:** Comparison of security features between our scheme and others.

	Ours	Qi *et al*.’s [14]	Lin *et al*.’s [21]	Mo *et al*.’s [22]
Provide credible mutual authentication	Y	Y	Y	Y
Provide anonymity	Y	N	Y	Y
Provide forward secrecy	Y	N	Y	N
Provide password update	Y	Y	N	N
Resist stolen-verifier attack	Y	-	Y	N
Resist smart card loss attack	Y	N	N	N
Resist denial-of-service attack	Y	Y	Y	Y
Resist impersonation attack	Y	N	Y	N
Resist off-line guessing attack	Y	N	Y	Y
Resist replay attack	Y	N	Y	Y

Since the registration phase just needs to be executed only once for a certain user, the password update phase isn’t always executed and the login and authentication phase is executed each time, we focus on the performance of login and authentication phase.

The running time of a hash function is *T*_*h*_, the running time of a large number addition is *T*_*LA*_, the running time of an elliptic curve scalar point multiplication is *T*_*PM*_ and the running time of a XOR operation is *T*_*x*_. Table 4 shows theoretical computational costs comparisons between the proposed scheme and other related schemes in the login and authentication phases. As we know, point multiplication based on elliptic curves is quite time-consuming operation [23–26], our proposed scheme and Lin *et al*.′*s* scheme have a great advantage on computational costs, because only hash, XOR and string connection operations are adopted in our scheme and Lin *et al*.′*s* scheme.

**Table 4:** Performance comparison in login and authentication phase.

scheme	User Computation	Server Computation	Total(ms)
Ours	7*T*_*x*_ + 8*T*_*h*_	3*T*_*x*_ + 4*T*_*h*_	10*T*_*x*_ + 12*T*_*h*_
Qi *et al*.’s [14]	5*T*_*x*_ + 6*T*_*h*_ + 2*T*_*PM*_	3*T*_*x*_ + 2*T*_*h*_ + *T*_*PM*_	8*T*_*x*_ + 10*T*_*h*_ + 3*T*_*PM*_
Lin *et al*.’s [21]	5*T*_*x*_ + 4*T*_*h*_	7*T*_*x*_ + 5*T*_*h*_ + 2*T*_*LA*_	12*T*_*x*_ + 9*T*_*h*_ + 2*T*_*LA*_
Mo *ea al*.’s [22]	*T*_*x*_ + 3*T*_*h*_ + 2*T*_*PM*_	*T*_*x*_ + 4*T*_*h*_ + 2*T*_*PM*_	2*T*_*x*_ + 7*T*_*h*_ + 4*T*_*PM*_

**Fig 5:**
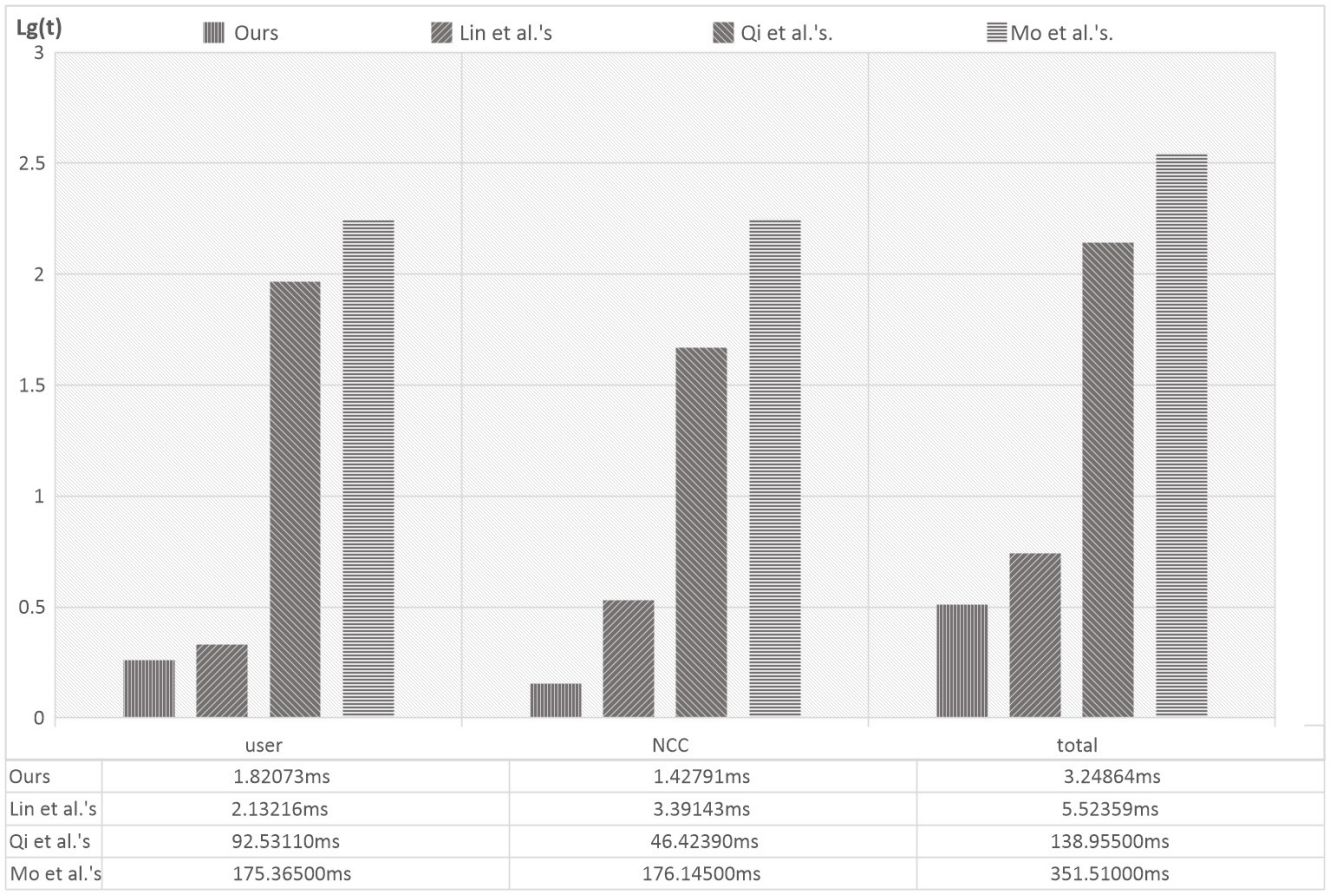
Computation cost comparisons of the login and authentication phase(the vertical axis is *lg*(*t*)).

We carried out a simulation of these schemes with Miracl Library. The hardware platform for user and sever is given an Inter(R) Core(TM) i7-6700 HQ CPU @ 2.60GHz and 8.00GB memory. The length of the random number we used in our simulation is 1000 bits, each simulation was performed for 100 times and the logarithm of average execute time(ms) in the login and authentication phase are shown in Fig 5. The bottom part of the Fig 5 shows real average execute time(ms) of the login and authentication phase. Because the value of time is too small, in the vertical axis of the histogram, we use *lg*(*t*) to represent the result, where *t* is real average execute time(ms). The computational costs in Qi *et al*.′*s* [14] and Mo *et al*.′*s* [22] schemes are much higher than our proposed scheme and Lin *et al*.′*s* [21] scheme. The reason is that those schemes involve point operations of an elliptic curve [27–30]. Thus, they are not suitable for satellite communication systems due to the limited computational capability of the devices. Compared with our scheme, the computational overhead of Lin *et al*.′*s* scheme costs a little more and we offer more security features such as anonymity, which is not provided in Lin *et al*.′*s* scheme. Therefore, our proposed scheme provides an efficient and secure authentication for satellite communication systems.

## Conclusions

In this paper, we proposed an efficient and provably secure key agreement scheme for satellite communication systems to provide credible mutual authentication. A dynamic temporary identity mechanism was adopted to ensure users’ anonymity. Besides, the traditional verification table was replaced by a dynamic verification table to resist denial-of-service attack caused by inconsistent data updating between *NCC* and user *U*. In addition, our scheme only adopted lightweight hash and string operations, which reduced the computational cost in comparison with other related works. We also proved the proposed scheme is provably secure in the real-or-random model. Therefore, the proposed scheme can meet the efficiency demands and security needs of communication satellite systems successfully.

## Supporting information

S1 File (DOCX)
