# Supplementary material for: An efficient and provably secure key agreement scheme for satellite communication systems
Source: PLoS One. 2021 Apr 26;16(4):e0250205. doi: 10.1371/journal.pone.0250205 (PMC8075218; doi:10.1371/journal.pone.0250205)
Supplement: S1 File — (DOCX) [file pone.0250205.s001.docx]

BYTES.h

#pragma once

#ifndef _BYTES

#define _BYTES

#define nam(value) #value //得到变量名称

extern "C" {

#include "miracl.h"

#include "mirdef.h"

}

#include "iostream"

#include "list"

#include "time.h"

using namespace std;

class BYTES { //一个BYTES对象可能只充当 big类型的作用 或者 BYTE数组的作用

private : //BYTES型只最大支持10进制5000位数的大数系统

big bigNum;

list<BYTE> bigNumBYTE;

bool bigChanged; //记录big是否变化 true变化 false未变化

bool BYTEChanged; //记录BYTE是否变化 true变化 false未变化

static bool isIniRandSeed; //是否初始化随机数种子

static bool isIniMiracl; //是否初始化大数系统

static int bit; //系统随机数位数

static void Irand() { //初始化随机数种子

if (!isIniMiracl) {

irand((long)time(NULL));

isIniRandSeed = true;

}

}

static void iMip() { //初始化大数系统

if (!isIniMiracl) {

mirsys(5000, 10);

isIniMiracl = true;

}

}

const big& getBigNum(); //得到该比特串

const list<BYTE> & getBYTE(); //得到该大数

public :

BYTES(); //空构造函数

BYTES(const BYTES&); //复制构造函数

BYTES(const big&); //big

BYTES(const list<BYTE>&); //字节型

BYTES(const string &);

~BYTES(); //析构函数

const list<BYTE>&getB()const {

return bigNumBYTE;

}

static void setBit(int b) {

bit = b;

}

BYTES operator+(const BYTES&);

BYTES operator|(const BYTES&);

BYTES operator^(const BYTES&);

BYTES operator=(const BYTES&);

bool operator==(const BYTES&);

void getRandom(); //得到一个随机素数

void getPrimeRandom(); //得到一个随机素数

string toString();

friend BYTES h(const BYTES &be);

friend ostream& operator <<(ostream &, BYTES &);

friend istream& operator>>(istream &, BYTES &);

};

#endif // !1

BYTES.cpp

#include "stdafx.h"

#include "BYTES.h"

#if _MSC_VER>=1900

#include "stdio.h"

_ACRTIMP_ALT FILE* __cdecl __acrt_iob_func(unsigned);

#ifdef __cplusplus

extern "C"

#endif

FILE* __cdecl __iob_func(unsigned i) {

return __acrt_iob_func(i);

}

#endif /* _MSC_VER>=1900 */

BYTES BYTES::operator+(const BYTES& b) { //判断是否初始化大数系统

big c = mirvar(0);

add(getBigNum(), b.bigNum, c);

return BYTES(c);

}

BYTES BYTES::operator|(const BYTES& b) { //待测

list<BYTE> c, d = getBYTE();

c.insert(c.begin(), d.begin(), d.end());

c.insert(c.end(), b.bigNumBYTE.begin(), b.bigNumBYTE.end());

return BYTES(c);

}

BYTES BYTES::operator^(const BYTES& b) {

list<BYTE> res; //位数少的高位补足0再异或-------------!

getBYTE();

if (bigNumBYTE.size() == b.bigNumBYTE.size()) {

int size = bigNumBYTE.size();

list<BYTE>::const_iterator iter1 = bigNumBYTE.begin(), iter2 = b.bigNumBYTE.begin();

for (int i = 0; i < size; ++i, ++iter1, ++iter2) {

res.push_back((*iter1) ^ (*iter2));

}

return BYTES(res);

}

const list<BYTE>* pMax = &b.bigNumBYTE, *pMin = &bigNumBYTE; //大小不等的情况下

if (bigNumBYTE.size() > b.bigNumBYTE.size()) {

pMax = &bigNumBYTE;

pMin = &b.bigNumBYTE;

}

list<BYTE> minClone = *pMin;

int offset = pMax->size() - pMin->size();

for (int i = 0; i < offset; ++i) {

minClone.push_front(BYTE(0)); //少几个0高位补几个0

}

//得到相同长度的BYTE串，再做异或操作

int size = pMax->size();

list<BYTE>::const_iterator iter1 = pMax->begin(), iter2 = minClone.begin();

for (int i = 0; i < size; ++i, ++iter1, ++iter2) {

res.push_back((*iter1) ^ (*iter2));

}

return BYTES(res);

}

bool BYTES::operator==(const BYTES& b) { //位数少的高位补足0再比较-------------!

getBYTE();

if (bigNumBYTE.size() == b.bigNumBYTE.size()) {

return bigNumBYTE == b.bigNumBYTE;

}

const list<BYTE>* pMax = &b.bigNumBYTE, *pMin = &bigNumBYTE; //大小不等的情况下

if (bigNumBYTE.size() > b.bigNumBYTE.size()) {

pMax = &bigNumBYTE;

pMin = &b.bigNumBYTE;

}

list<BYTE> minClone = *pMin;

int offset = pMax->size() - pMin->size();

for (int i = 0; i < offset; ++i) {

minClone.push_front(BYTE(0)); //少几个0高位补几个0

}

return minClone == *pMax;

}

BYTES BYTES::operator=(const BYTES& b) {

bigChanged = b.bigChanged;

BYTEChanged = b.BYTEChanged;

copy(b.bigNum, bigNum); //复制B

bigNumBYTE = b.bigNumBYTE;

return *this;

}

ostream& operator <<(ostream &out, BYTES &data) {

data.getBYTE();

out << data.bigChanged << " " << data.BYTEChanged << endl;

out << data.bigNumBYTE.size() << endl;

for (list<BYTE>::iterator iter = data.bigNumBYTE.begin(); iter != data.bigNumBYTE.end(); ++iter) {

out << (int)*iter << " ";

}

out << endl;

return out;

}

istream& operator>>(istream &ins, BYTES &data) {

data.bigNumBYTE.clear();

int size;

int ch;

ins >> data.bigChanged >> data.BYTEChanged;

ins >> size;

for (int i = 0; i < size; ++i) {

ins >> ch;

data.bigNumBYTE.push_back((BYTE)ch);

}

return ins;

}

BYTES h(const BYTES &b) {

sha hash;

shs_init(&hash);

char aim[20];

for (list<BYTE>::const_iterator iter = b.bigNumBYTE.begin(); iter != b.bigNumBYTE.end(); ++iter) {

shs_process(&hash, *iter);

}

shs_hash(&hash, aim);

BYTES temp;

for (int i = 0; i < 20; ++i) {

temp.bigNumBYTE.push_back(aim[i]);

}

temp.BYTEChanged = true; //标志位改变

return temp;

}

void BYTES::getRandom() {

big a = mirvar(0);

bigbits(BYTES::bit, a);

(*this) = BYTES(a);

mirkill(a); //释放内存

getBYTE();

}

void BYTES::getPrimeRandom() {

big a = mirvar(0), b = mirvar(0);

bigbits(BYTES::bit, a);

nxprime(a, b);

(*this) = BYTES(b);

mirkill(a);

mirkill(b); //释放内存

getBYTE();

}

BYTES::BYTES(const string &s) {

iMip();

Irand();

bigNum = mirvar(0); //分配空间

for (int i = 0; i < (int)s.length(); ++i) {

bigNumBYTE.push_back(s[i]);

}

bigChanged = false;

BYTEChanged = true;

}

BYTES::BYTES() {

iMip();

Irand();

bigNum = mirvar(0); //分配空间

bigChanged = false; //标致位

BYTEChanged = false; //标致位

}

BYTES::BYTES(const BYTES& b) {

iMip();

Irand();

bigNum = mirvar(0); //分配空间

bigNumBYTE = b.bigNumBYTE;

copy(b.bigNum, bigNum); //复制B

bigChanged = b.bigChanged; //标致位

BYTEChanged = b.BYTEChanged; //标致位

}

BYTES::BYTES(const big& b) {

iMip();

Irand();

bigNum = mirvar(0); //分配空间

copy(b, bigNum); //复制B

bigChanged = true; //标致位

BYTEChanged = false; //标致位

}

BYTES::BYTES(const list<BYTE>& b) {

iMip();

Irand();

bigNum = mirvar(0); //分配空间

this->bigNumBYTE = b;

bigChanged = false;

BYTEChanged = true;

}

BYTES::~BYTES() {

mirkill(bigNum);

bigNumBYTE.clear();

}

const list<BYTE> & BYTES::getBYTE() {

if (bigChanged) { //如果big被改变过 重新转换

int _bit;

if (BYTES::bit % 8 == 0) //得到最大字节

_bit = BYTES::bit / 8;

else

_bit = BYTES::bit / 8 + 1;

char *bytes = new char[_bit];

big_to_bytes(_bit, bigNum, bytes, true); //将big转换为Bytes

for (int i = 0; i < _bit; ++i) {

bigNumBYTE.push_back(bytes[i]);

}

delete[]bytes; //释放内存

bigChanged = false; //转换完毕，标志位改变

BYTEChanged = false;

}

return bigNumBYTE;

}

const big& BYTES::getBigNum() {

if (BYTEChanged) { //若BYTE改变过 重新转换

BYTE *bytes = new BYTE[bigNumBYTE.size()];

list<BYTE>::const_iterator iter = bigNumBYTE.begin(); int i = 0;

for (; iter != bigNumBYTE.end(); ++iter, ++i) {

bytes[i] = (BYTE)*iter;

}

bytes_to_big(bigNumBYTE.size(), (char*)bytes, bigNum);

delete[]bytes;

bigChanged = false; //转换完毕，标志位改变

BYTEChanged = false;

}

return bigNum;

}

string BYTES::toString() {

string output;

char ch[3];

list<BYTE>::const_iterator iter = bigNumBYTE.begin();

for (; iter!=bigNumBYTE.end(); ++iter)

{

sprintf_s(ch, 3, "%02X", (BYTE)*iter);

output += ch;

}

output += '\n';

return output;

}

bool BYTES::isIniRandSeed = false; //是否初始化随机数种子

bool BYTES::isIniMiracl = false; //是否初始化大数系统

int BYTES::bit = 1000; //系统随机数位数

CDialog.h

#include "scheme.h"

// CDialog2 对话框

class CDialog2 : public CDialog

{

DECLARE_DYNAMIC(CDialog2)

private :

scheme_Ini *ncc=NULL;

scheme_Reg *reg=NULL;

scheme_Authen *au=NULL;

public:

CWnd *userEdit;

CWnd *nccEdit;

CDialog2(CWnd* pParent = nullptr); // 标准构造函数

string GetID();

string GetPW();

int GetRadio();

void SetID(LPCTSTR s);

void SetPW(LPCTSTR s);

virtual ~CDialog2();

// 对话框数据

#ifdef AFX_DESIGN_TIME

enum { IDD = IDD_DIALOG2 };

#endif

protected:

virtual void DoDataExchange(CDataExchange* pDX); // DDX/DDV 支持

DECLARE_MESSAGE_MAP()

public:

afx_msg void OnBnClickedAu();

// afx_msg void OnBnClickedButtonTt();

afx_msg void OnBnClickedIni();

afx_msg void OnBnClickedDisplay();

afx_msg void OnBnClickedReg();

// afx_msg void OnBnClickedClear();

};

// CDialog2.cpp: 实现文件

//

#include "stdafx.h"

#include "sessionKeyGenerateTool.h"

#include "CDialog2.h"

#include "afxdialogex.h"

// CDialog2 对话框

IMPLEMENT_DYNAMIC(CDialog2, CDialog)

CDialog2::CDialog2(CWnd* pParent /*=nullptr*/)

: CDialog(IDD_DIALOG2, pParent)

{

}

void CDialog2::SetID(LPCTSTR s) {

GetDlgItem(IDC_EDIT_AU_ID)->SetWindowTextW(s);

}

void CDialog2::SetPW(LPCTSTR s){

GetDlgItem(IDC_EDIT_AU_PW)->SetWindowTextW(s);

}

string CDialog2::GetID() {

CString s;

GetDlgItem(IDC_EDIT_AU_ID)->GetWindowText(s);

return string(CT2CA(s));

}

string CDialog2::GetPW() {

CString s;

GetDlgItem(IDC_EDIT_AU_PW)->GetWindowText(s);

return string(CT2CA(s));

}

int CDialog2::GetRadio() {

CButton *b1, *b2,*b3;

b1 = (CButton*)GetDlgItem(IDC_RADIO1);

b2 = (CButton*)GetDlgItem(IDC_RADIO2);

b3 = (CButton*)GetDlgItem(IDC_RADIO3);

if (b1->GetCheck())

return 1;

else if (b2->GetCheck())

return 2;

else if (b3->GetCheck())

return 3;

}

CDialog2::~CDialog2()

{

}

void CDialog2::DoDataExchange(CDataExchange* pDX)

{

CDialog::DoDataExchange(pDX);

}

BEGIN_MESSAGE_MAP(CDialog2, CDialog)

ON_BN_CLICKED(ID_AU, &CDialog2::OnBnClickedAu)

// ON_BN_CLICKED(IDC_BUTTON_TT, &CDialog2::OnBnClickedButtonTt)

ON_BN_CLICKED(ID_INI, &CDialog2::OnBnClickedIni)

ON_BN_CLICKED(ID_DISPLAY, &CDialog2::OnBnClickedDisplay)

ON_BN_CLICKED(ID_REG, &CDialog2::OnBnClickedReg)

//ON_BN_CLICKED(ID_CLEAR, &CDialog2::OnBnClickedClear)

END_MESSAGE_MAP()

// CDialog2 消息处理程序

void CDialog2::OnBnClickedAu()

{

userEdit->SetWindowText(_T(""));

nccEdit->SetWindowText(_T(""));

if (GetID() == string("")) {

MessageBox(_T("账号不能为空"), _T("提示"), MB_ICONINFORMATION); //检测编辑框状态、

return;

}

else if (GetPW() == string("")) {

MessageBox(_T("密码不能为空"), _T("提示"), MB_ICONINFORMATION);

return;

}

if (GetRadio() == 0) {

MessageBoxW(_T("选择不能为空"), _T("提示"), MB_ICONINFORMATION);

return;;

}

BYTES ID(GetID());

BYTES PW(GetPW());

au = new scheme_Authen(reg->sc, reg->db);

if (GetRadio() == 1) {

au->isAttack1 = true;

}else

if (GetRadio() == 2) {

au->isAttack2 = true;

}

else {

au->isAttack2 = false;

au->isAttack1 = false;

}

au->excute(ID, PW);

SetID(_T(""));

SetPW(_T(""));

if (au->_SK.getB().empty()) {

MessageBox(_T("相互认证失败"), _T("提示"), MB_ICONERROR);

return;

}

MessageBox(_T("相互认证成功"), _T("提示"), MB_ICONINFORMATION);

}

void CDialog2::OnBnClickedIni() {

int result;

CButton *pB1 = (CButton*)GetDlgItem(ID_REG), *pB2 = (CButton*)GetDlgItem(ID_AU);

if (!ncc) {

ncc = ncc = new scheme_Ini();

pB1->ShowWindow(true);

pB2->ShowWindow(false);

SetID(_T(""));

SetPW(_T(""));

MessageBoxW(_T("初始化NCC完毕"), _T("提示"), MB_ICONINFORMATION);

return;

}

result = MessageBoxW(_T("是否重新初始化NCC"), _T("提示"), MB_ICONQUESTION | MB_YESNO);

switch (result)

{

case IDYES:

delete ncc;

ncc = new scheme_Ini();

pB1->ShowWindow(true);

pB2->ShowWindow(false);

SetID(_T(""));

SetPW(_T(""));

MessageBoxW(_T("初始化NCC完毕"), _T("提示"), MB_ICONINFORMATION);

break;

case IDNO:

return;

break;

}

// TODO: 在此添加控件通知处理程序代码

}

void CDialog2::OnBnClickedDisplay()

{

if (!ncc) {

MessageBoxW(_T("NCC尚未被初始化"), _T("提示"), MB_ICONINFORMATION);

return;

}

out::addNCC(CString("\r\nNCC密钥："));

out::addNCC(CString(ncc->x.toString().c_str()));

out::addNCC(CString("\r\n"));

}

void CDialog2::OnBnClickedReg()

{

//注册用户

if (!ncc) {

MessageBoxW(_T("NCC尚未被初始化"), _T("提示"), MB_ICONINFORMATION);

return;

}

if (GetID().empty()) {

MessageBoxW(_T("注册账号不能为空"), _T("提示"), MB_ICONINFORMATION);

return;

}

if (GetPW().empty()) {

MessageBoxW(_T("注册密码不能为空"), _T("提示"), MB_ICONINFORMATION);

return;

}

BYTES ID (GetID());

BYTES PW (GetPW());

reg = new scheme_Reg(*ncc);

reg->excute(ID, PW);

CButton *pB1 = (CButton*)GetDlgItem(ID_REG),*pB2= (CButton*)GetDlgItem(ID_AU);

pB1->ShowWindow(false);

pB2->ShowWindow(true);

MessageBoxW(CString("注册完成\r\n账号:")+CString(GetID().c_str())+CString("\r\n")+CString("密码:")+CString(GetPW().c_str()) + CString("\r\n"), _T("提示"), MB_ICONINFORMATION);

SetID(_T(""));

SetPW(_T(""));

}

认证协议头文件

scheme.h

#ifndef SCHEME_FILE

#define SCHEME_FILE

#include "BYTES.h"

#include "iostream"

using namespace std;

class out {

public :

static CWnd *userE;

static CWnd *nccE;

static void addNCC(CString );

static void addUser(CString );

};

class scheme_Ini { //方案初始化类

public:

BYTES x;

scheme_Ini() {

x.getPrimeRandom();

out::addUser(CString((string("生成: ") + x.toString() + string("\r\n")).c_str()));

}

};

class scheme_smartCard {

public:

BYTES Tid, R, r1, verify, Z;

};

class scheme_DataBase {

public:

BYTES hID, Tid, Tid0, hPW;

};

class scheme_Reg { //方案注册类

private:

scheme_Ini ncc;

BYTES r0, hPW, PW, ID, r1, P, x, R, Tid, Tid0, verify, Z, hID;

scheme_Reg() {};

public:

scheme_smartCard sc; //通过id找到SC 默认拥有SC

scheme_DataBase db;

void excute(BYTES& ID, BYTES& PW);

scheme_Reg(scheme_Ini n) {

ncc = n;

}

};

class scheme_Authen {

private:

scheme_DataBase db;

scheme_smartCard sc;

BYTES ID, PW, _r0, _verify, _P, r1, r2, Q, S, _hID, Tid, __P, x, _S, _r2, r3, Tidnew, v1, v2, v3, _r3, _Tidnew, _v2, verify, R, Z, hID, hPW, SK;

scheme_Authen() {}

public:

bool isAttack1 = false;

bool isAttack2 = false;

scheme_Authen(scheme_smartCard s, scheme_DataBase db) {

sc = s;

this->db = db;

//初始化SC中的数据到认证阶段

Tid = s.Tid;

r1 = s.r1;

R = s.R;

verify = s.verify;

Z = s.Z;

hID = db.hID;

hPW = db.hPW;

}

void excute(BYTES& ID, BYTES& PW);

BYTES _SK;

};

#endif // !1

认证协议实现

scheme.cpp

#include "stdafx.h"

#include "scheme.h"

#include "string"

CWnd* out::userE;

CWnd* out::nccE;

void scheme_Reg::excute(BYTES& ID, BYTES& PW) {

r0.getRandom(); //生成r0

hPW = h(PW ^ r0);

out::addUser(CString((string("计算: ") + hPW.toString() + string("\r\n")).c_str()));

out::addUser(CString(string("User 向 NCC 发出注册请求\r\n").c_str()));

hID = h(ID^r0);

out::addNCC(CString((string("计算: ") + hID.toString() + string("\r\n")).c_str()));

r1.getRandom();

P = h(hID | x);

out::addNCC(CString((string("计算: ") + P.toString() + string("\r\n")).c_str()));

R = P ^ h(hID | r1);

out::addNCC(CString((string("计算: ") + R.toString() + string("\r\n")).c_str()));

Tid.getRandom(); //生成Tid，Tid0默认为0

//下面存SmartCard

sc.Tid = Tid;

sc.R = R;

sc.r1 = r1;

//下面存Database

db.hID = hID;

db.hPW = hPW;

db.Tid = Tid;

db.Tid0 = Tid0;

verify = h(ID | r0 | PW | r1);

out::addUser(CString((string("计算: ") + verify.toString() + string("\r\n")).c_str()));

Z = r0 ^ h(PW | ID);

out::addUser(CString((string("计算: ") + Z.toString() + string("\r\n")).c_str()));

//下面存SmartCard

sc.verify = verify;

sc.Z = Z;

out::addUser(CString("生成SmartCard:{\r\n"));

out::addUser(CString((string("Tid: ")+Tid.toString()+string(",\r\n")).c_str()));

out::addUser(CString((string("R: ") + R.toString() + string(",\r\n")).c_str()));

out::addUser(CString((string("r1: ") + r1.toString() + string(",\r\n")).c_str()));

out::addUser(CString((string("Z: ") + Z.toString() + string(",\r\n")).c_str()));

out::addUser(CString((string("verify: ") + verify.toString() + string("\r\n}\r\n\r\n")).c_str()));

}

void scheme_Authen::excute(BYTES& ID, BYTES& PW) {

//User

_SK = BYTES();

_r0 = Z ^ h(PW | ID);

out::addUser(CString((string("计算: ") + _r0.toString() + string("\r\n")).c_str()));

_verify = h(ID | _r0 | PW | r1);

out::addUser(CString("\r\n第1次验证\r\n"));

out::addUser(CString("verify1:"));

out::addUser(CString(verify.toString().c_str()));

out::addUser(CString("verify1':"));

out::addUser(CString(_verify.toString().c_str()));

if (!(_verify == verify)) {

out::addUser(CString("\r\n账号或密码错误，验证中断\r\n"));

out::addNCC(CString("\r\n账号或密码错误，验证中断\r\n"));

return;

}

r2.getRandom();

_hID = h(ID ^ _r0);

out::addUser(CString((string("计算: ") + _hID.toString() + string("\r\n")).c_str()));

_P = R ^ h(_hID | r1);

out::addUser(CString((string("计算: ") + _P.toString() + string("\r\n")).c_str()));

Q = _P ^ r2^h(PW^_r0);

out::addUser(CString((string("计算: ") + Q.toString() + string("\r\n")).c_str()));

S = h(_hID | r2 | Tid);

out::addUser(CString((string("计算: ") + S.toString() + string("\r\n")).c_str()));

out::addUser(CString(string("User 向 NCC 发送数据\r\n").c_str()));

//NCC

__P = h(hID | x);

out::addNCC(CString((string("计算: ") + _P.toString() + string("\r\n")).c_str()));

_r2 = Q ^ __P^hPW;

if (isAttack1) {

out::addNCC(CString("r2数据被改动"));

_r2.getRandom();

}

out::addNCC(CString((string("计算: ") + _r2.toString() + string("\r\n")).c_str()));

_S = h(hID | _r2 | Tid);

out::addNCC(CString("\r\n第2次验证\r\n"));

out::addNCC(CString("verify2:"));

out::addNCC(CString(S.toString().c_str()));

out::addNCC(CString("verify2':"));

out::addNCC(CString(_S.toString().c_str()));

if (!(_S == S)) {

out::addUser(CString("\r\n由User传向NCC的数据被篡改,验证中断\r\n"));

out::addNCC(CString("\r\n由User传向NCC的数据被篡改,验证中断\r\n"));

return;

}

r3.getRandom();

Tidnew.getRandom(); //把Tidnew当随机数处理 存Tid不变

v1 = __P ^ r3;

out::addNCC(CString((string("计算: ") + v1.toString() + string("\r\n")).c_str()));

v2 = h(__P | _r2 | r3 | Tidnew);

out::addNCC(CString((string("计算: ") + v2.toString() + string("\r\n")).c_str()));

v3 = h(v1 | _r2) ^ Tidnew;

out::addNCC(CString((string("计算: ") + v3.toString() + string("\r\n")).c_str()));

SK = h(hID | _r2 | r3 | __P);

out::addNCC(CString((string("计算: ") + SK.toString() + string("\r\n")).c_str()));

//User

_r3 = v1 ^ _P;

out::addUser(CString((string("计算: ") + _r3.toString() + string("\r\n")).c_str()));

_Tidnew = v3 ^ h(v1 | r2);

out::addUser(CString((string("计算: ") +Tidnew.toString() + string("\r\n")).c_str()));

_v2 = h(_P | r2 | _r3 | Tidnew);

if (isAttack2) {

out::addNCC(CString("r2数据被改动\r\n"));

_v2.getRandom();

}

out::addUser(CString((string("计算: ") + _v2.toString() + string("\r\n")).c_str()));

out::addUser(CString("\r\n第3次验证\r\n"));

out::addUser(CString("verify3:"));

out::addUser(CString(v2.toString().c_str()));

out::addUser(CString("\r\nverify3':"));

out::addUser(CString(_v2.toString().c_str()));

if (!(_v2 == v2)) {

out::addUser(CString("\r\n由NCC传向User的数据被篡改,验证中断\r\n"));

out::addNCC(CString("\r\n由NCC传向User的数据被篡改,验证中断\r\n"));

return;

}

_SK = h(_hID | r2 | _r3 | _P);

out::addUser(CString("\r\n认证成功\r\n会话密钥':"));

out::addNCC(CString("\r\n认证成功\r\n会话密钥:"));

out::addNCC(CString(SK.toString().c_str()));

out::addUser(CString(_SK.toString().c_str()));

isAttack1 = false;

isAttack2 = false;

}

void out::addNCC(CString s) {

CString cur;

nccE->GetWindowText(cur);

nccE->SetWindowText(cur + s);

Sleep(60);

nccE->UpdateWindow();

nccE->SendMessage(WM_VSCROLL, SB_BOTTOM, 0);

}

void out::addUser(CString s) {

CString cur;

userE->GetWindowText(cur);

userE->SetWindowText(cur + s);

Sleep(60);

userE->UpdateWindow();

userE->SendMessage(WM_VSCROLL, SB_BOTTOM, 0);

}

主界面头文件

// sessionKeyGenerateToolDlg.h: 头文件

//

#pragma once

#include "scheme.h"

#include "CDialog2.h"

// CsessionKeyGenerateToolDlg 对话框

class CsessionKeyGenerateToolDlg : public CDialogEx

{

// 构造

public:

CsessionKeyGenerateToolDlg(CWnd* pParent = nullptr); // 标准构造函数

CDialog2 *m_para2; //第二个Tab页

// 对话框数据

#ifdef AFX_DESIGN_TIME

enum { IDD = IDD_SESSIONKEYGENERATETOOL_DIALOG };

#endif

protected:

virtual void DoDataExchange(CDataExchange* pDX); // DDX/DDV 支持

// 实现

protected:

HICON m_hIcon;

// 生成的消息映射函数

virtual BOOL OnInitDialog();

afx_msg void OnSysCommand(UINT nID, LPARAM lParam);

afx_msg void OnPaint();

afx_msg HCURSOR OnQueryDragIcon();

DECLARE_MESSAGE_MAP()

public:

// afx_msg void OnBnClickedOk();

afx_msg void OnBnClickedButton1();

afx_msg void OnClose();

CTabCtrl m_tabCtr;

afx_msg void OnTcnSelchangeTab1(NMHDR *pNMHDR, LRESULT *pResult);

};

主界面实现

// sessionKeyGenerateToolDlg.cpp: 实现文件

//

#include "stdafx.h"

#include "sessionKeyGenerateTool.h"

#include "sessionKeyGenerateToolDlg.h"

#include "afxdialogex.h"

#include <conio.h>

using namespace std;

#ifdef _DEBUG

#define new DEBUG_NEW

#endif

// 用于应用程序“关于”菜单项的 CAboutDlg 对话框

class CAboutDlg : public CDialogEx

{

public:

CAboutDlg();

// 对话框数据

#ifdef AFX_DESIGN_TIME

enum { IDD = IDD_ABOUTBOX };

#endif

protected:

virtual void DoDataExchange(CDataExchange* pDX); // DDX/DDV 支持

// 实现

protected:

DECLARE_MESSAGE_MAP()

};

CAboutDlg::CAboutDlg() : CDialogEx(IDD_ABOUTBOX)

{

}

void CAboutDlg::DoDataExchange(CDataExchange* pDX)

{

CDialogEx::DoDataExchange(pDX);

}

BEGIN_MESSAGE_MAP(CAboutDlg, CDialogEx)

END_MESSAGE_MAP()

// CsessionKeyGenerateToolDlg 对话框

CsessionKeyGenerateToolDlg::CsessionKeyGenerateToolDlg(CWnd* pParent /*=nullptr*/)

: CDialogEx(IDD_SESSIONKEYGENERATETOOL_DIALOG, pParent)

{

m_hIcon = AfxGetApp()->LoadIcon(IDR_MAINFRAME);

}

void CsessionKeyGenerateToolDlg::DoDataExchange(CDataExchange* pDX)

{

CDialogEx::DoDataExchange(pDX);

DDX_Control(pDX, IDC_TAB1, m_tabCtr);

}

BEGIN_MESSAGE_MAP(CsessionKeyGenerateToolDlg, CDialogEx)

ON_WM_SYSCOMMAND()

ON_WM_PAINT()

ON_WM_QUERYDRAGICON()

// ON_BN_CLICKED(IDOK, &CsessionKeyGenerateToolDlg::OnBnClickedOk)

ON_WM_CLOSE()

ON_NOTIFY(TCN_SELCHANGE, IDC_TAB1, &CsessionKeyGenerateToolDlg::OnTcnSelchangeTab1)

END_MESSAGE_MAP()

// CsessionKeyGenerateToolDlg 消息处理程序

BOOL CsessionKeyGenerateToolDlg::OnInitDialog()

{

CDialogEx::OnInitDialog();

m_tabCtr.InsertItem(0, _T("初始化/注册/认证"));//“选项卡1”可更改，是sheet页的名字；

m_para2 = new CDialog2;

//m_para1->Create(IDD_DIALOG1, &m_tabCtr);

m_para2->Create(IDD_DIALOG2, &m_tabCtr);

out::userE = GetDlgItem(IDC_UserText);

out::nccE = GetDlgItem(IDC_NCCText);

m_para2->userEdit = out::userE;

m_para2->nccEdit = out::nccE;

CRect rs;

m_tabCtr.GetClientRect(&rs);//调整子对话框在父窗口中的位置，可以改动数值，使子窗体的大小合适；

rs.top+=26;

rs.bottom-=3;

rs.left+=2;

rs.right-=3;//设置子对话框尺寸并移动到指定位置

m_para2->MoveWindow(&rs);

//m_para1->ShowWindow(true);

m_para2->ShowWindow(true);

m_tabCtr.SetCurSel(0);

// 将“关于...”菜单项添加到系统菜单中。

// IDM_ABOUTBOX 必须在系统命令范围内。

ASSERT((IDM_ABOUTBOX & 0xFFF0) == IDM_ABOUTBOX);

ASSERT(IDM_ABOUTBOX < 0xF000);

CMenu* pSysMenu = GetSystemMenu(FALSE);

if (pSysMenu != nullptr)

{

BOOL bNameValid;

CString strAboutMenu;

bNameValid = strAboutMenu.LoadString(IDS_ABOUTBOX);

ASSERT(bNameValid);

if (!strAboutMenu.IsEmpty())

{

pSysMenu->AppendMenu(MF_SEPARATOR);

pSysMenu->AppendMenu(MF_STRING, IDM_ABOUTBOX, strAboutMenu);

}

}

// 设置此对话框的图标。 当应用程序主窗口不是对话框时，框架将自动

// 执行此操作

SetIcon(m_hIcon, TRUE); // 设置大图标

SetIcon(m_hIcon, FALSE); // 设置小图标

// TODO: 在此添加额外的初始化代码

return TRUE; // 除非将焦点设置到控件，否则返回 TRUE

}

void CsessionKeyGenerateToolDlg::OnSysCommand(UINT nID, LPARAM lParam)

{

if ((nID & 0xFFF0) == IDM_ABOUTBOX)

{

CAboutDlg dlgAbout;

dlgAbout.DoModal();

}

else

{

CDialogEx::OnSysCommand(nID, lParam);

}

}

// 如果向对话框添加最小化按钮，则需要下面的代码

// 来绘制该图标。 对于使用文档/视图模型的 MFC 应用程序，

// 这将由框架自动完成。

void CsessionKeyGenerateToolDlg::OnPaint()

{

if (IsIconic())

{

CPaintDC dc(this); // 用于绘制的设备上下文

SendMessage(WM_ICONERASEBKGND, reinterpret_cast<WPARAM>(dc.GetSafeHdc()), 0);

// 使图标在工作区矩形中居中

int cxIcon = GetSystemMetrics(SM_CXICON);

int cyIcon = GetSystemMetrics(SM_CYICON);

CRect rect;

GetClientRect(&rect);

int x = (rect.Width() - cxIcon + 1) / 2;

int y = (rect.Height() - cyIcon + 1) / 2;

// 绘制图标

dc.DrawIcon(x, y, m_hIcon);

}

else

{

CDialogEx::OnPaint();

}

}

//当用户拖动最小化窗口时系统调用此函数取得光标

//显示。

HCURSOR CsessionKeyGenerateToolDlg::OnQueryDragIcon()

{

return static_cast<HCURSOR>(m_hIcon);

}

void CsessionKeyGenerateToolDlg::OnClose()

{

// TODO: 在此添加消息处理程序代码和/或调用默认值

CDialogEx::OnClose();

}

void CsessionKeyGenerateToolDlg::OnTcnSelchangeTab1(NMHDR *pNMHDR, LRESULT *pResult)

{

// TODO: 在此添加控件通知处理程序代码

*pResult = 0;

m_para2->ShowWindow(false);

int CurSel = m_tabCtr.GetCurSel();

m_para2->ShowWindow(true);

}

工程头文件

// sessionKeyGenerateTool.h: PROJECT_NAME 应用程序的主头文件

//

#pragma once

#ifndef __AFXWIN_H__

#error "在包含此文件之前包含“stdafx.h”以生成 PCH 文件"

#endif

#include "resource.h" // 主符号

// CsessionKeyGenerateToolApp:

// 有关此类的实现，请参阅 sessionKeyGenerateTool.cpp

//

class CsessionKeyGenerateToolApp : public CWinApp

{

public:

CsessionKeyGenerateToolApp();

// 重写

public:

virtual BOOL InitInstance();

// 实现

DECLARE_MESSAGE_MAP()

};

extern CsessionKeyGenerateToolApp theApp;

工程实现

// sessionKeyGenerateTool.cpp: 定义应用程序的类行为。

//

#include "stdafx.h"

#include "sessionKeyGenerateTool.h"

#include "sessionKeyGenerateToolDlg.h"

#ifdef _DEBUG

#define new DEBUG_NEW

#endif

// CsessionKeyGenerateToolApp

BEGIN_MESSAGE_MAP(CsessionKeyGenerateToolApp, CWinApp)

ON_COMMAND(ID_HELP, &CWinApp::OnHelp)

END_MESSAGE_MAP()

// CsessionKeyGenerateToolApp 构造

CsessionKeyGenerateToolApp::CsessionKeyGenerateToolApp()

{

// 支持重新启动管理器

m_dwRestartManagerSupportFlags = AFX_RESTART_MANAGER_SUPPORT_RESTART;

// TODO: 在此处添加构造代码，

// 将所有重要的初始化放置在 InitInstance 中

}

// 唯一的 CsessionKeyGenerateToolApp 对象

CsessionKeyGenerateToolApp theApp;

// CsessionKeyGenerateToolApp 初始化

BOOL CsessionKeyGenerateToolApp::InitInstance()

{

// 如果一个运行在 Windows XP 上的应用程序清单指定要

// 使用 ComCtl32.dll 版本 6 或更高版本来启用可视化方式，

//则需要 InitCommonControlsEx()。 否则，将无法创建窗口。

INITCOMMONCONTROLSEX InitCtrls;

InitCtrls.dwSize = sizeof(InitCtrls);

// 将它设置为包括所有要在应用程序中使用的

// 公共控件类。

InitCtrls.dwICC = ICC_WIN95_CLASSES;

InitCommonControlsEx(&InitCtrls);

CWinApp::InitInstance();

AfxEnableControlContainer();

// 创建 shell 管理器，以防对话框包含

// 任何 shell 树视图控件或 shell 列表视图控件。

CShellManager *pShellManager = new CShellManager;

// 激活“Windows Native”视觉管理器，以便在 MFC 控件中启用主题

CMFCVisualManager::SetDefaultManager(RUNTIME_CLASS(CMFCVisualManagerWindows));

// 标准初始化

// 如果未使用这些功能并希望减小

// 最终可执行文件的大小，则应移除下列

// 不需要的特定初始化例程

// 更改用于存储设置的注册表项

// TODO: 应适当修改该字符串，

// 例如修改为公司或组织名

SetRegistryKey(_T("应用程序向导生成的本地应用程序"));

CsessionKeyGenerateToolDlg dlg;

m_pMainWnd = &dlg;

INT_PTR nResponse = dlg.DoModal();

if (nResponse == IDOK)

{

// TODO: 在此放置处理何时用

// “确定”来关闭对话框的代码

}

else if (nResponse == IDCANCEL)

{

// TODO: 在此放置处理何时用

// “取消”来关闭对话框的代码

}

else if (nResponse == -1)

{

TRACE(traceAppMsg, 0, "警告: 对话框创建失败，应用程序将意外终止。\n");

TRACE(traceAppMsg, 0, "警告: 如果您在对话框上使用 MFC 控件，则无法 #define _AFX_NO_MFC_CONTROLS_IN_DIALOGS。\n");

}

// 删除上面创建的 shell 管理器。

if (pShellManager != nullptr)

{

delete pShellManager;

}

#if !defined(_AFXDLL) && !defined(_AFX_NO_MFC_CONTROLS_IN_DIALOGS)

ControlBarCleanUp();

#endif

// 由于对话框已关闭，所以将返回 FALSE 以便退出应用程序，

// 而不是启动应用程序的消息泵。

return FALSE;

}
